# Investigating Gene–Gene and Gene–Environment Interactions in the Association Between Overnutrition and Obesity-Related Phenotypes

**DOI:** 10.3389/fgene.2019.00151

**Published:** 2019-03-04

**Authors:** François Tessier, Bénédicte Fontaine-Bisson, Jean-François Lefebvre, Ahmed El-Sohemy, Marie-Hélène Roy-Gagnon

**Affiliations:** ^1^School of Epidemiology and Public Health, University of Ottawa, Ottawa, ON, Canada; ^2^School of Nutrition Sciences, University of Ottawa, Ottawa, ON, Canada; ^3^Department of Nutritional Sciences, University of Toronto, Toronto, ON, Canada

**Keywords:** genetic, gene–environment interactions, overnutrition, obesity, BMI, macronutrient, IKKβ/NF-κB

## Abstract

**Introduction:** Animal studies suggested that *NFKB1*, *IKBKB*, and *SOCS3* genes could be involved in the association between overnutrition and obesity. This study aims to investigate interactions involving these genes and macronutrient intakes affecting obesity-related phenotypes.

**Methods:** We used a traditional statistical method, logistic regression, and compared it to alternative statistical method, multifactor dimensionality reduction (MDR) and penalized logistic regression (PLR), to better detect genes/environment interactions in the Toronto Nutrigenomics and Health Study (*n* = 1639) using dichotomized body mass index (BMI) and waist circumference as obesity-related phenotypes. Exposure variables included genotype on 54 single nucleotide polymorphisms (*NFKB1*: 18, *IKBKB*: 9, *SOCS3*: 27), macronutrient (carbohydrates, protein, fat) and alcohol intakes and ethno-cultural background.

**Results:** After correction for multiple testing, no interaction was found using logistic regression. MDR identified interactions between SOCS3 rs6501199 and rs4969172, and *IKBKB* rs3747811 affecting BMI in the Caucasian population; SOCS3 rs6501199 and *NFKB1* rs1609798 affecting WC in the Caucasian population; and SOCS3 rs4436839 and IKBKB rs3747811 affecting WC in the South Asian population. PLR found a main effect of SOCS3 rs12944581 on BMI among the South Asian population.

**Conclusion:** While MDR and PLR had discordant results, some models support results from previous studies. These results emphasize the need to use alternative statistical methods to investigate high-order interactions and suggest that variants in the nutrient-responsive hypothalamic IKKB/NF-kB signaling pathway may be involved in obesity pathogenesis.

## Introduction

During the last decades, the obesity epidemic has been a major concern for public health. An estimated four million deaths worldwide are due to excess of body weight in 2015 (The GBD 2015 Obesity Collaborators, 2017). In addition to sedentary lifestyle, which is a well-known factor in obesity pathogenesis, diet also has a major role in the development of body weight excess, particularly due to the high accessibility of energy-dense foods (Swinburn et al., 2011).

Animal studies on mice might have found a new lead on how eating habits could influence the regulation of hunger and the risk of developing body weight excess (Zhang et al., 2008; Meng and Cai, 2011; Benzler et al., 2015; Dalvi et al., 2017; Douglass et al., 2017). More specifically, they found that overnutrition achieved by giving a chronic high fat diet as well as an acute overload of glucose activated the IKKβ/NF-κB signaling pathway in the hypothalamus, the central structure regulating energy homeostasis (Zhang et al., 2008; Douglass et al., 2017). The hypothalamic IKKβ/NF-κB signaling pathway is involved in the inflammatory response, and contributes to the pathogenesis of obesity by inducing an insulin and leptin resistance through inflammation of the hypothalamic cells (Lee et al., 1996; Obici et al., 2002; Zhang et al., 2008; Douglass et al., 2017). The hypothalamic resistance to these hormones is also known to be associated with appetite dysregulation, increased hunger, and neuronal inflammation (Lehrke and Lazar, 2004; Hotamisligil, 2006; Zhang et al., 2008; Douglass et al., 2017). However, this association has not been yet observed in humans.

This study aimed to investigate this phenomenon in human subjects by exploring possible gene–gene and gene–environment interactions of genes involved in the IKKβ/NF-κB signaling pathway and macronutrient (carbohydrate, protein, and fat) and alcohol intakes. The three genes of interest are *nuclear factor kappa B subunit 1* (*NFKB1*), *inhibitor of kappa light polypeptide gene enhancer in B-cells, kinase beta* (*IKBKB*), and *suppressor of cytokine signaling 3* (*SOCS3*). *NFKB1* codes for the subunit 1 of the NF-κB protein complex, and *IKBKB* codes for the IKKβ protein that phosphorylates the inhibitor of the NF-κB complex, allowing it to be activated (Arkan et al., 2005; Zhang et al., 2008). Mice with astrocyte-specific deletion of *IKKβ* in the mediobasal hypothalamus have been shown to have reduced susceptibility to high fat diet induced hypothalamic inflammation, and thus are at lower risk of diet induced obesity (Douglass et al., 2017). The downstream gene of the IKKβ/NF-κB hypothalamic signaling pathway, *SOCS3*, codes for a protein of the same name acting as an inhibitor of insulin and leptin signaling (Howard and Flier, 2006; Zhang et al., 2008). Only *SOCS3* has previously been investigated in humans, and few studies have found evidence of an association with obesity-related phenotypes (Talbert et al., 2009; Tang et al., 2011). Although, multiple studies have investigated gene–gene and gene–environment interactions involved in obesity pathogenesis (Ordovás et al., 2011; Reddon et al., 2016; Rask-Andersen et al., 2017; Mangum and Mangum, 2018), no study has yet investigate potential interactions involving *NFKB1, IKBKB, SOCS3* and macronutrients and alcohol intakes.

We hypothesized that polymorphisms in genes involved in the hypothalamic IKKβ/NF-κB signaling pathway (*NFKB1*, *IKBKB*, and *SOCS3*), alone or in interaction with nutrients, are associated with energy imbalance and obesity pathogenesis in humans. Furthermore, since standard statistical methods have difficulties handling high order interactions or lack power to detect their effect, we hypothesize that these interactions may be better detected using alternative approaches (Cordell, 2009; Thomas, 2010) such as multifactor dimensionality reduction (MDR) (Ritchie and Motsinger, 2005) and penalized logistic regression (PLR) (Park and Hastie, 2008).

## Materials and Methods

### Study Population

Participants were young adults aged 20–29 years participating in the Toronto Nutrigenomics and Health Study, a multiethnic cohort composed of 1,639 participants. They were recruited through postings and advertisement around the area of the University of Toronto campus between September 2004 and July 2009. Participants completed a general health and lifestyle questionnaire to assess physical activity levels, smoking habits, and sociodemographic characteristics. Caloric, macronutrients, and alcohol intakes were estimated using a 196-item semi-quantitative food frequency questionnaire (García-Bailo et al., 2012). Individuals who may have underreported (<800 kcal/day) or overreported (>3,500 kcal/day for women, >4,000 kcal/day for men) their daily energy intakes were excluded (*n* = 124). These exclusion criteria are based on plausible intakes for this age group as previously described in the Toronto Nutrigenomics and Health Study. Participants with missing data for the outcome variables were also excluded (*n* = 3). Thus, after exclusions, 1,512 participants remained in the sample (1,033 women and 479 men).

An open-ended question was used to determine the participants’ ethnocultural status. Based on their self-reported status, they were categorized into four ethnocultural groups: 733 Caucasians (237 men and 496 women), 509 East Asians (142 men and 367 women), 160 South Asians (65 men and 95 women), and 110 others (35 men and 75 women). Caucasians included European, Middle Eastern, and Hispanic. East Asians were composed of Chinese, Japanese, Korean, Filipino, Vietnamese, Thai, and Cambodian. South Asians comprised Bangladeshi, Indian, Pakistani, and Sri Lankan. The “other” group was composed of participants belonging to ≥2 of the four ethnocultural groups, or First Nations Canadians or Afro-Caribbeans.

### Dietary Assessment and Lifestyle Variables

The average monthly food consumption was calculated using a semi-quantitative 196-items Willett food frequency questionnaire (García-Bailo et al., 2012). Participants were given instructions on how to complete the food frequency questionnaire, and an example of a commonly used portion size (e.g., half a cup) was given to each item. Then, daily intakes of carbohydrates, fat, protein and alcohol were estimated in kilocalories using the USDA Nutrient Database for Standard Reference. By combining all the macronutrients and alcohol intakes into daily intakes, a total calorie intake was also estimated for each participant. As proposed by Willett and Stampfer (1986), we adjusted each environmental variables (macronutrients and alcohol) for total energy intake by using the residual of the regression of macronutrient and alcohol on total caloric intake, since energy intake is associated with obesity-related phenotypes.

The general health and lifestyle questionnaire was used to assess the physical activity levels – quantified as modifiable metabolic equivalent of task (MET) hours per week – and the smoking status of the participants (current smoker or non-smoker) (García-Bailo et al., 2012).

### Anthropometric Measurements and Outcome Variables

The two outcomes of interest (waist circumference and BMI) were both measured by trained personnel with participants dressed in light clothing without shoes (García-Bailo et al., 2012). Waist circumference was measured between the lowest rib and iliac crest and was measured twice to the nearest 0.1 cm. A third measurement was taken when the difference between the two measurements was ≥1 cm, and the two measurements with the smallest difference were taken to calculate the mean waist circumference. Weight was measured to the nearest 0.1 kg using a digital scale (model Bellissima 841, Seca Corporation, Hanover, MD, United States), and height was measured to the nearest 0.1 cm using a wall-mounted stadiometer (model Seca 206, Seca Corporation, Hanover, MD, United States). Subsequently, BMI (kg/m^2^) was calculated for each participant.

We dichotomized BMI and waist circumference into high and low categories. The dichotomized BMI was based on the cut-off points recommended by the National Institutes of Health [NIH] (1998) and World Health Organization [WHO] (2008), which yielded a high BMI group composed of participants considered as overweight and obese (BMI ≥ 25.0 kg/m^2^), and a low BMI group composed of participants with normal BMI and underweight (BMI < 25.0 kg/m^2^). For waist circumference, the definition of high waist circumference proposed by WHO (≥102 cm for men, ≥88 cm for women) was not used because few participants were eligible to be categorize into the high waist circumference group (World Health Organization [WHO], 2008) perhaps due to the young age of the participants. Hence, we used the last quartile of the waist circumference distribution to define the high waist circumference group, stratified by sex. Therefore, the high waist circumference group was defined as waist circumference ≥85.25 cm for men, and ≥74.73 cm for women. Otherwise participants were considered to have a low waist circumference.

Since it is now recognized that standard BMI threshold might not be appropriate for individuals of East Asian and South Asian ethnicity, an alternate BMI definition was used to make a comparison with the standard BMI definition in a sensitivity analysis. This alternate BMI definition uses the dichotomized BMI threshold stated before for white individuals but categorizes East Asian and South Asian individuals in the high BMI group if BMI ≥ 23.0 kg/m^2^, and others were categorized in the low BMI group. This alternate BMI is based on a WHO expert consultation, which recommended BMI cut-off points for the Asian population of 23.0 kg/m^2^ ≤ BMI < 27.5 kg/m^2^ for overweight individuals, and BMI ≥ 27.5 kg/m^2^ for obese (World Health Organization [WHO], 2004). However, WHO experts recommend to still use the current WHO BMI cut-off points, particularly in countries with concurrent problems of undernutrition and overnutrition, since the new cut-off points are still up to debate, and more research is needed on this matter.

### Selection of Tagging SNPs and Genotyping

Tag SNP selection was made using HapMap release 27 and Haploview 4.2, with a minimum minor allele frequency of 5% and *r*^2^ threshold of 0.80 using pairwise tagging (De Bakker et al., 2005). For quality control, 10% of the population was genotyped a second time and a >99% concordance was achieved. An initial 54 single-nucleotide polymorphisms (SNPs) were selected among the three genes of interest: 27 for *SOCS3*, 18 for *NFKB1*, and 9 for *IKBKB*. First, we tested for Hardy-Weinberg equilibrium (HWE). All tag SNPs were in HWE (HWE test *p*-value > 0.01). Afterward, we excluded 30 SNPs with minor allele frequency < 5%. Finally, four SNPs were excluded because they were in high linkage disequilibrium (*R*^2^ > 0.8). The final SNPs selection was eight for *SOCS3*, nine for *NFKB1*, and three for *IKBKB*. SNPs effects were coded as an additive effect for logistic regression models, but were considered as discrete variables for MDR (since it can only handle discrete variables) and PLR (Park and Hastie, 2008).

### Statistical Analysis

Three methods were used to analyze the data. We use a standard statistical method, logistic regression, which was then compared to two alternative statistical methods: MDR and PLR.

Firstly, we used logistic regression to investigate twofold gene–gene interactions, and twofold gene–environment interactions. Logistic regression models for gene–gene interaction models were adjusted for age, sex and ethnocultural background, while gene–environment interaction models were adjusted for age, physical activity, ethnocultural background and sex.

Secondly, we used MDR to analyze twofold and threefold gene–gene and gene–environment interactions. This technique was developed by Ritchie et al. (2001) to detect high order gene–gene and gene–environment interactions in common genetic diseases for a dichotomous outcome. MDR is considered a non-parametric and model-free method that transforms a high-multilocus model (including genetic and/or environmental factors) to a one dimensional model (Ritchie et al., 2001). Succinctly, this method aims to find the best interaction order and the best set of factors that determine a disease dichotomous status. The MDR algorithm starts with a 10-fold cross-validation for each possible set of factors to determine the best set of factors. In this step, MDR divides the dataset into a training part (9/10 of the data) and a testing part (1/10 of the data). For all combinations of factors, it uses the training part to create contingency tables based on cases and controls. Then, subjects in cells with a cases/controls ratio greater than 1 are labeled as high-risk, while the other cells are labeled low-risk. Based on this new categorization of the training part (9/10), the training error is calculated. With the testing part (1/10), the prediction error is calculated. These steps are repeated for each cross-validation fold (by default, a 10-fold cross-validation). Then, the prediction error and the cross-validation consistencies – how many times a best set of factors was selected in the 10-fold cross-validation) are compared. The selection of the best MDR model is done by selecting the model with the smallest prediction error and/or the largest cross-validation consistency. In this study, we considered an MDR model valid if it had an average testing (1 – prediction error) accuracy ≥55% and if the cross-validation consistency of a model was >6/10.

Thirdly, we used the PLR developed by Park and Hastie (2008), a parametric approach using a L2 regularization to detect gene–gene interactions. The L2 regularization is used to reduce overfitting problems by preventing the model weights from becoming too small or too large (Goeman, 2010; Goeman et al., 2014). This method includes a forward stepwise procedure variable selection (Park and Hastie, 2008; He et al., 2009). The final model is the one with the smallest score C. This score is C = deviance + cp ×*df*. Cp is the complexity parameter, which is by default 2. *Df* is the effective degrees of freedom.

Multifactor dimensionality reduction and PLR include model selection procedures and do not rely on individual *p*-values to select significant interactions. Hence, these methods do not require a separate procedure to account for multiple comparisons. For our standard logistic regression analyses, we used the false discovery rate (FDR) approach to account for multiple comparisons (Storey, 2011).

## Results

Subject characteristics based on their dichotomized BMI and waist circumference are provided in Table 1. Because the sample had twice as many women as men, we were expecting about 66% of women in each group. However, the high BMI group was roughly composed of 50% women, while the low BMI group had a significant difference in sex proportion, with 73% women Participants from the high BMI group were slightly older on average [age difference (±standard deviation) = 0.56 (±2.5) years, *p* = 0.018]. No difference was found in smoking status between the two groups. The proportions of participants with high BMI among the different ethnocultural groups were 56% Caucasians, 19% East Asians, 14% South Asians, and 11% others (*p* < 0.001). The proportion of subjects with a high BMI varied highly within each ethnocultural backgrounds: 25% of Caucasians, 12% of East Asians, 29% of South Asians, and 34% of others. The two BMI groups were similar in terms of education (data not shown), which was expected since participants were recruited around the area of the University of Toronto campus, and physical activity levels (*p* > 0.05). Total calorie, fat, protein, and alcohol intakes were higher for the high BMI group then the low BMI group (*p* < 0.05). No difference in the characteristics of the high and low waist circumference groups was statistically significant, except for the ethnocultural background (*p* < 0.001). The participants characteristics of the high and low alternative BMI cut-offs are shown in Table 2. These cut-offs adapted for Asian populations resulted in more participants classified in the high BMI group. There were also significant differences (*p* < 0.001) between the high and low alternative BMI groups observed for sex, ethnocultural background, protein intakes and alcohol intakes (*p* < 0.05).

**Table 1:** General characteristics of study participants by high/low BMI and high/low waist circumference categories (*n* = 1,512).

	High BMI (*n* = 323)	Low BMI (*n* = 1,189)	*p*-value	High waist circumference (*n* = 376)	Low waist circumference (*n* = 1,136)	*p*-value
Female	163 (50.5)	870 (73.2)	<0.001	257 (68.4)	776 (68.3)	1.000
Age (years)	23.1 (2.7)	22.6 (2.4)	0.018	23.0 (2.6)	22.6 (2.5)	0.239
Smoking	29 (9.0)	69 (5.8)	0.054	24 (6.4)	74 (6.5)	1.000
Ethnocultural group			<0.001			<0.001
Caucasians	180 (55.7)	553 (46.5)		226 (60.1)	507 (44.6)	
East Asian	60 (18.6)	449 (37.8)		72 (19.2)	437 (38.5)	
South Asian	46 (14.2)	114 (9.6)		48 (12.8)	112 (9.9)	
Others	37 (11.5)	73 (6.1)		30 (8.0)	80 (7.0)	
METS	6.3 (2.3)	6.2 (2.5)	0.233	6.3 (2.4)	6.2 (2.5)	0.333
Total calories (kcal/day)	2,003.6 (698.6)	1,960.8 (637.1)	0.033	1,956.7 (647.1)	1,974.3 (652.1)	0.867
Carbohydrates (kcal/day)	259.1 (99.6)	259.9 (92.1)	0.073	253.7 (92.5)	261.7 (94.1)	0.707
Fats (kcal/day)	67.1 (28.4)	65.3 (26.1)	0.047	66.00 (27.9)	65.6 (26.1)	0.119
Proteins (kcal/day)	88.2 (35.8)	84.7 (31.8)	0.007	85.6 (32.3)	85.3 (32.9)	0.697
Alcohol (kcal/day)	7.2 (11.6)	5.3 (8.5)	<0.001	6.5 (9.3)	5.4 (9.3)	0.896

*Data are n (%) or mean (standard deviation). P-values are from T-tests and chi-squared tests. Low BMI: <25.0 kg/m^2^; high BMI: ≥25.0 kg/m^2^. For men low waist circumference: <85.25 cm; high waist circumference: ≥85.25 cm. For women low waist circumference: <74.73 cm; high waist circumference: ≥74.73 cm.*

**Table 2:** General characteristics of study participants using alternate BMI cut-offs (*n* = 1,512).

	High BMI (*n* = 444)	Low BMI (*n* = 1,068)	*p*-value
Female	236 (53.2)	797 (74.6)	<0.001
Age (years)	22.8 (2.6)	22.7 (2.5)	0.060
Smoke	33 (7.4)	65 (6.1)	0.358
Race			<0.001
Caucasians	180 (40.5)	553 (51.8)	
East Asian	152 (34.2)	357 (33.4)	
South Asian	75 (16.9)	85 (8.0)	
Other	37 (8.3)	73 (6.8)	
METS	6.2 (2.4)	6.3 (2.5)	0.578
Total calories (kcal/day)	1,995.7 (685.8)	1,959.2 (635.6)	0.053
Carbohydrates (kcal/day)	261.0 (97.9)	259.2 (92.0)	0.116
Fats (kcal/day)	65.9 (27.3)	65.6 (26.3)	0.327
Proteins (kcal/day)	89.0 (35.9)	83.9 (31.2)	<0.001
Alcohol (kcal/day)	5.8 (10.3)	5.63 (8.9)	<0.001

*Data are n (%) or mean (standard deviation). P-values are from T-tests and chi-squared tests. For East and South Asian populations, low alternative BMI: <23.0 kg/m^2^; high alternative BMI: ≥23.0 kg/m^2^.*

Using logistic regression, none of the twofold gene–gene and gene–environment interaction models were statistically significant for BMI, alternative BMI and waist circumference after adjustment for multiple testing with FDR. Logistic regression with significant p-values before adjustment with FDR are shown in Table 3.

**Table 3:** Gene–gene interaction logistic regression models.

Outcome	Gene–gene	SNP–SNP	Wald test *p*-value	FDR *q*-value
BMI	SOCS3–NFKB1	rs4436839–rs3774932	0.037	0.332
		rs4436839–rs1599961	0.013	0.291
		rs4436839–rs3774956	0.022	0.291
		rs4436839–rs11722146	0.005	0.291
		rs4436839–rs4698863	0.040	0.338
		rs4436839–rs1609798	0.007	0.291
		rs6501199–rs3774932	0.022	0.291
		rs6501199–rs11722146	0.011	0.291
		rs6501199–rs3774968	0.035	0.332
		rs6501199–rs7674640	0.030	0.332
		rs6501199–rs4698863	0.022	0.291
		rs6501199–rs1609798	0.004	0.291
		rs12944581–rs3774932	0.037	0.332
		rs12944581–rs1599961	0.017	0.291
Waist circumference	SOCS3–NFKB1	rs4436839–rs11722146	0.041	0.597
		rs4436839–rs1609798	0.032	0.597
		rs6501199–rs3774932	0.023	0.597
		rs6501199–rs11722146	0.019	0.597
		rs6501199–rs3774968	0.034	0.597
		rs6501199–rs7674640	0.038	0.597
		rs6501199–rs4698863	0.018	0.597
		rs6501199–rs1609798	0.006	0.597
Alternative BMI	SOCS3–NFKB1	rs9914220–rs3774956	0.032	0.469

*Adjusted for age, sex, and ethnocultural background. FDR, false discovery rate.*

With MDR, we looked at the best twofold and threefold interaction models for each outcome of interest (BMI, alternative BMI, waist circumference) including all the participants. A stratified analysis followed based on the ethnocultural background: Caucasians only, East Asians only, South Asians only. We used the quartiles of the macronutrients and alcohol residuals, based on ethnocultural background and sex, to evaluate the gene–environment interactions with MDR.

**FIGURE 1:**
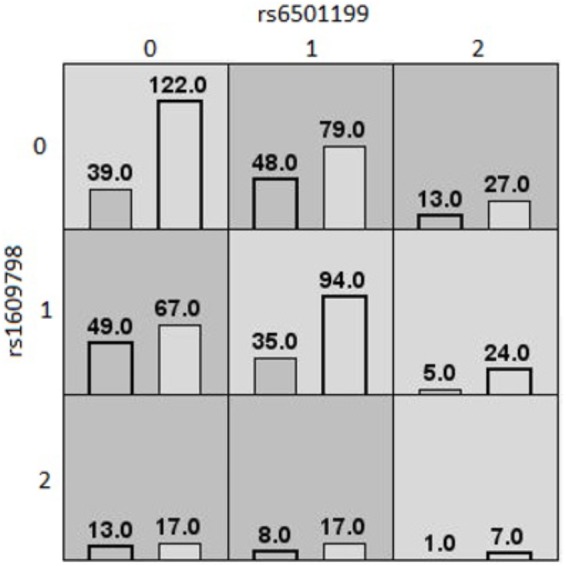
MDR contingency table of the best twofold gene–gene interaction model of high/low waist circumference (Caucasians). MDR contingency tables of the twofold gene–gene interaction model for waist circumference considering only Caucasians. The interaction is composed of rs6501199 (*SOCS3*) and rs160978 (*NFKB1*). Each cell represents a specific combination of both factors, where “0” represents individuals homozygous for the common allele, “1” the heterozygous, and “2” the homozygous for the rare allele. The number of individuals fitting of a specific combination of factors is represented by bars within the cells. The left bar is the count of cases (high waist circumference), and the right bar is the count of controls (low waist circumference). Dark gray cells are cells that have been identified as “high risk” by MDR, and the others are identified as “low risk” cells.

Table 4 shows the three MDR models that were selected as valid models, based on an average testing accuracy ≥55%, and a cross-validation consistency ≥6/10. The first MDR model was found in the Caucasian population. It was a twofold interaction model for the waist circumference outcome composed of rs6501199 (SOCS3) and rs160978 (NFKB1), with an average testing accuracy of 55% and a cross-validation consistency of 7/10. Figure 1 shows the MDR graphical representation of this model, where individuals with one or two rare alleles of rs160978 (*NFKB1*) are identified by MDR to be more likely to have a high waist circumference if they have the common alleles for rs6501199 (*SOCS3*). The opposite effect can be observed for Caucasians with one or two rare alleles of rs6501199 (*SOCS3*) in the presence of the common alleles of rs160978 (*NFKB1*). Heterozygotes and homozygotes for the rare alleles of rs160978 (*NFKB1*) and rs6501199 (*SOCS3*) are categorized by MDR to be less likely to have a high waist circumference, except for individuals who are homozygotes for the rare allele of rs160978 (*NFKB1*) and are heterozygotes for rs6501199 (*SOCS3*).

**Table 4:** MDR models for twofold and threefold interactions.

Population	Outcome	Model	Average testing accuracy	CV consistency
Caucasian	Waist circumference	rs6501199 (*SOCS3*), rs1609798 (*NFKB1*)	55%	7/10
Caucasian	BMI	rs6501199 (*SOCS3*), rs4969172 (*SOCS3*), rs3747811 (*IKBKB*)	60%	10/10
South Asian	Waist circumference	rs4436839 (*SOCS3*), rs3747811 (*IKBKB*)	55%	8/10

*The average testing accuracy is the proportion of individuals that are correctly classified as being a case or a control, averaged across all CV intervals. The CV consistency is the number of times a model is identified in each possible 9/10 of the data.*

**FIGURE 2:**
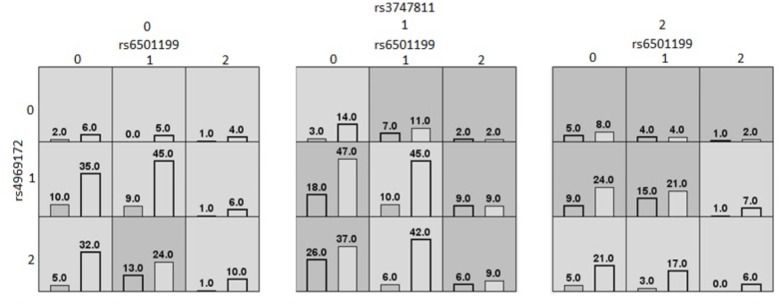
MDR contingency table of the threefold gene–gene interaction model of high/low BMI (Caucasians). MDR contingency tables of the threefold gene–gene interaction model considering only Caucasians. The SNPs involved are rs6501199 (*SOCS3*), rs4969172 (*SOCS3*), and rs3747811 (*IKBKB*). Each cell represents a specific combination of SNPs alleles, where “0” represents individuals homozygous for the common allele, “1” the heterozygous, and “2” the homozygous for the rare allele. The number of individuals fitting of a specific genotype is represented by bars within the cells. The left bar is the count of cases (high BMI)_;_ and the right bar is the count of controls (low BMI). Dark gray cells are cells that have been identified as “high risk” by MDR, and the others are identified as “low risk” cells.

The second MDR model represented in Figure 2, was also found in the Caucasian population. It was a threefold interaction model for the BMI outcome composed of rs6501199 (SOCS3), rs4969172 (SOCS3), and rs3747811 (IKBKB), with an average testing accuracy of 60% and cross-validation consistency of 10/10. This MDR model suggests Caucasians with the one or two alleles for rs3747811 (IKBKB) are more generally likely to have high BMI, depending of the combination of alleles for rs6501199 (SOCS3) and rs4969172 (SOCS3). Although all homozygotes for the common allele for rs3747811 (IKBKB) are evaluated to be at low risk of having a high BMI, this is not true for Caucasians heterozygotes for rs4969172 (SOCS3) and homozygotes for rs6501199 (SOCS3). Heterozygotes for rs3747811 (IKBKB) are identified to be more likely to have high BMI when in the presence of one or two rare alleles for the two other SNPs involved in this model, except for Caucasians heterozygotes or homozygotes for the rare allele of rs4969172 (SOCS3) who are also heterozygotes for rs6501199 (SOCS3). As for the homozygotes for the rare allele of rs3747811 (IKBKB), they are generally more likely to have high BMI, according to MDR, except for Caucasians who are homozygotes for the rare allele of rs4969172 (SOCS3), and for heterozygotes for rs4969172 (SOCS3) who also are homozygotes for the rare allele of rs6501199 (SOCS3).

**FIGURE 3:**
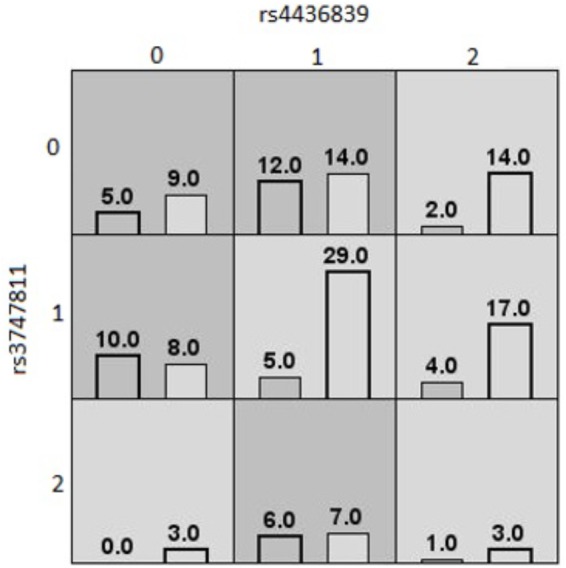
MDR contingency table of the best twofold gene–gene interaction model of high/low waist circumference (South Asians). MDR contingency tables of the twofold gene–gene interaction model for waist circumference considering only South Asians. The interaction is composed of rs3747811 (*IKBKB*) and rs4436839 (*SOCS3*). Each cell represents a specific combination of both factors, where “0” represents individuals homozygous for the common allele, “1” the heterozygous, and “2” the homozygous for the rare allele. The number of individuals fitting of a specific combination of factors is represented by bars within the cells. The left bar is the count of cases (high waist circumference), and the right bar is the count of controls (low waist circumference). Dark gray cells are cells that have been identified as “high risk” by MDR, and the others are identified as “low risk” cells.

The last MDR model, shown in Figure 3, was found in the South Asian population. This model was a twofold interaction model assessing high or low waist circumference composed of rs3747811 (*IKBKB*) and rs4436839 (*SOCS3*), with an average testing accuracy of 55% and a cross-validation of 8/10. This model suggests that South Asians who are homozygote for the two SNPs involved are more likely to have high waist circumference. Additionally, individuals with one rare allele for one of these two SNPs are also considered to be at high risk of having an increased waist circumference. The other genotypes are less likely to have a high waist circumference, except for heterozygotes for rs4436839 (*SOCS3*) who are homozygote for the rare allele of rs3747811 (*IKBKB*). None of the gene–environment MDR models had an average testing accuracy ≥55%, and a cross-validation consistency ≥6/10. Therefore, no gene–environment MDR model was considered as valid.

With the Park and Hastie PLR method, models stratified by ethnocultural background detected no interaction. Only null models were selected by this method, except for the South Asian population, where the gene–gene and the gene–environment PLR models for the alternative BMI both selected rs12944581 (*SOCS3*). This model suggests that individuals with the common genotype for rs12944581 (*SOCS3*) have higher odds of having a high alternative BMI (*p* < 0.001), while heterozygous (*p* = 0.322) and homozygous (*p* = 0.088) for rs12944581 (*SOCS3*) rare allele seems to reduce the odds of high alternative BMI.

## Discussion

In this study, we investigated gene–gene and gene–environment interactions in the association between variants of three genes (*NFKB1*, *IKBKB, SOCS3*), macronutrient and alcohol intakes, and obesity-related phenotypes (BMI, alternative BMI cut-offs for East and South Asians, and waist circumference). These three genes are involved in the hypothalamic IKKβ/NF-κB/SOCS3 signaling pathway, which has been previously identified to play an important role in obesity pathogenesis through its activation by overnutrition in mice (Zhang et al., 2008). The activation of the hypothalamic IKKβ/NF-κB signaling pathway disrupts the normal hypothalamic regulation of satiety and hunger. The objective of this study was to investigate this phenomenon in humans using logistic regression (a traditional statistical method) and alternative statistical methods, such as PLR and MDR.

With standard MDR, we detected three different interactions. Two of these models were detected among the Caucasian population. One was a twofold gene–gene interaction model involving rs6501199 (*SOCS3*) and rs1609798 (*NFKB1*) on the outcome of high or low waist circumference. The first MDR model found among the Caucasian population was a twofold interaction model for the waist circumference outcome composed of rs6501199 (SOCS3) and rs160978 (NFKB1). Although, there is no study that has investigated the involvement of rs160978 (*NFKB1*), this MDR model suggests that the rare allele of rs160978 (*NFKB1*) may increase the activation of the NF-κB complex, which may contribute to an increased risk of obesity-related phenotypes in the presence of the wild type genotype (i.e., two common alleles) for rs6501199 (*SOCS3*). However, the rare allele of rs6501199 (*SOCS3*) seems to interact by generally canceling the deleterious effect of the rs160978 (*NFKB1*) rare allele. Hence, the SOCS3 protein, which is downstream of the hypothalamic IKKβ/NF-κB pathway, of an individual heterozygous or homozygous for the rare allele would be harder to activate.

The second MDR model, represented in Figure 2, found among the Caucasian population suggested a threefold gene–gene interaction between rs6501199 (*SOCS3*), rs4969172 (*SOCS3*), and rs3747811 (*IKBKB*) on the outcome of high or low BMI. Of these three SNPs, only rs6501199 (*SOCS3*) had been found to be weakly associated with visceral adipose tissue in a previous study (Talbert et al., 2009). In the same study, rs4969172 (*SOCS3*) was also investigated for association with multiple obesity-relate traits phenotypes (including BMI, and waist circumference), but none was found. As for rs3747811 (*IKBKB*), it has not been yet investigated in previous studies for its involvement in the development of obesity-related phenotypes, and was only found to decrease the risk of colorectal cancer combined with rs4648110 (*NFKB1*) (Seufert et al., 2013). A possible biological interpretation is that the rare allele of rs3747811 (*IKBKB*) triggers the activation of the NF-κB complex, thus also inducing *SOCS3* expression, resulting in insulin/leptin resistance. The two other SNPs in this 3-way interaction (rs6501199 and rs4969172) are part of the same gene, *SOCS3*. By looking at the MDR model contingency table their interpretation is less clear, but these two *SOCS3* polymorphisms or other unmeasured polymorphisms in high LD might modify the coded protein that contributes to insulin/leptin resistance.

The last MDR model found was among the South Asian population. It suggested a twofold interaction between rs4436839 (*SOCS3*) and rs3747811 (*IKBKB*) on the outcome of high or low waist circumference, where the common alleles of both SNPs seem to contribute to potentially through a better activation of the NF-κB complex activity by the IKBKB protein and increased insulin/leptin resistance by the SOCS3 protein. However, this MDR model is less reliable on its biological interpretation, since several cells have low frequencies (particularly cells for the homozygous of the rare allele of rs3747811).

Although, we did not find any interaction with PLR through the forward stepwise selection procedure proposed by Park and Hastie (2008), a main effect of rs12944581 (*SOCS3*) on the alternative BMI was found among the South Asian population, suggesting that individuals with the common genotype had higher odds of being in the high alternative BMI group. This SNP has been previously investigated in a Hispanic cohort, but no association with obesity-related traits (including BMI and waist circumference) was found.

The MDR and PLR models did not provide convergent results except for the involvement of the SOCS3 gene. With logistic regression before adjustment for multiple testing, we found one model that was similar to the MDR model involving a twofold interaction between rs6501199 (SOCS3) and rs1609798 (NFKB1) on waist circumference. However, these similar models did not have the same interpretation. While the rs6501199 heterozygous the common genotype for rs1609798 have higher odds of having a high waist circumference according to the logistic regression model, the opposite relationship can be seen in the MDR model. Hence, we cannot confirm that MDR support the logistic regression model.

The alternative statistical methods that we considered – MDR and PLR – did not detect any interaction involving environmental factors, based on our MDR criteria or on PLR’s forward stepwise selection procedure. Although it could suggest that there is no gene–environment interaction between diet and *SOCS3*, *IKBKB*, and *NFKB1*, it could also be explained by the four levels categorization of macronutrient intake we used with MDR or a poor assessment of macronutrient intakes by the FFQ (Kipnis et al., 2003; Howard and Flier, 2006).

A strength of this study is the use of alternative statistical methods, which enabled us to detect interaction that would not have been detected with traditional logistic regression. In addition, this study is one of the first to investigate gene–gene and gene–environment interactions between *NFKB1*, *IKBKB, SOCS3* and macronutrient intakes on human and their effect on obesity-related phenotypes. Our results will help to guide future research on this pathway.

Several potential limitations need to be acknowledged. First, we had a limited sample size to detect interactions. We thus chose to use methods like MDR and PLR that were specifically developed to circumvent the lack of power offered by traditional methods like logistic regression with usual multiple comparison correction. Estimating power for MDR or the PLR is not straightforward and would require extensive simulations, which were outside the scope of this study. The power of these methods was investigated previously (Edwards et al., 2009; He et al., 2009; Molinaro et al., 2011) and greatly depends on the disease model. However, these studies show that sample of sizes comparable to ours would allow the detection of interactions in realistic situations. We used the QUANTO software (Gauderman, 2002; Gauderman and Morrison, 2009) to estimate the power of our largest sub-sample (Caucasians) to detect a range of effect sizes in a logistic regression context, setting our significance level to 0.000035 to account for multiple comparisons. For gene–gene interactions among the 54 SNPs considered, we estimated that we had over 80% power to detect an interaction odds ratio (OR) of 1.7 or more, with remaining parameter values based on those observed in our data. We had approximately 30–50% power using logistic regression to detect an interaction OR of 1.4–1.5, which is in the range of values observed for the interaction models detected by MDR. For gene–environment interactions, with parameter values based on those observed in our data, we had over 80% power to detect an interaction OR of 1.05 or greater, indicating that we had good power to detect gene–environment interactions.

Other limitations included the fact that participants were young (20–29 years) and educated, due to the recruitment taking part through advertisement on the University of Toronto campus. These characteristics might explain why few participants were obese, since young adults are less overweight compared to adults aged more than 35 years, and educated individuals in high-income countries are usually less likely to be obese (Cohen et al., 2013). In addition, the multi-ethnic nature of the participants introduced problems of population heterogeneity. Stratification by ethno-cultural background, needed for adjustment, drastically reduced the number of participants in the MDR and PLR models. The subjective definitions used to categorize participants into an ethno-cultural group might not represent accurately their ethno-cultural background and does not consider possible population stratification of sub-ethnicity within one category. In addition, given our limited sample size, we chose not to assess interactions with sex in addition to gene–gene or gene–nutrient intake interactions. Effect modification by sex would need to be investigated in future studies, ideally with larger sample size. Lastly, an important limitation is the utilization of the FFQ for the assessment of macronutrient and alcohol intakes. FFQs have been found to be prone to important measurement errors, which has created a fair number of debates in the field of nutritional epidemiology (Subar et al., 2015). Ultimately, replication of our results in an independent sample will be required to confirm the potential interactions found in our study.

## Conclusion

In conclusion, interaction involving gene part of the hypothalamic IKKβ/NF-κB signaling pathway were identified using alternative statistical methods – MDR and PLR. However, both methods found different models, and no gene–environment interaction was found with macronutrient and alcohol intakes. Because of this discrepancy, these results should be carefully interpreted. Further investigation is needed in order to determine whether any effects are real. However, this study is the first to investigate gene–gene and gene–environment interactions between polymorphism in *NFKB1*, *IKBKB*, *SOCS3*, macronutrient and alcohol intakes, in the association between overnutrition and obesity-related phenotypes in human subjects. Hence, our findings offer suggestions for future investigation of this phenomenon and contribute to the understanding of the role of insulin and leptin resistance in obesity pathogenesis in humans.

## Ethics Statement

This project was approved by the Ottawa Health Science Network Research Ethics Board and from the University of Toronto.

## Author Contributions

M-HR-G and BF-B designed the project with input from FT. The Toronto Nutrigenomics and Health study database was accessed through a collaboration with AE-S (University of Toronto). FT conducted all analyses and wrote the manuscript. J-FL provided guidance on the use of the required specialized statistical software. All authors reviewed and approved the final manuscript.

## Conflict of Interest Statement

The authors declare that the research was conducted in the absence of any commercial or financial relationships that could be construed as a potential conflict of interest.
